# Adrenocortical Response to Stress and Thyroid Hormone Status in Free-Living Nestling White Storks (*Ciconia ciconia*) Exposed to Heavy Metal and Arsenic Contamination

**DOI:** 10.1289/ehp.9099

**Published:** 2006-07-11

**Authors:** Raquel Baos, Julio Blas, Gary R. Bortolotti, Tracy A. Marchant, Fernando Hiraldo

**Affiliations:** 1 Department of Applied Biology, Estación Biológica de Doñana, Sevilla, Spain; 2 Department of Biology, University of Saskatchewan, Saskatoon, Saskatchewan, Canada

**Keywords:** Aznalcóllar, corticosterone, free-living birds, handling-restraint protocol, lead, metal pollution, stress response, thyroxine, triiodothyronine, white stork

## Abstract

**Background/Objective:**

Endocrine parameters have proven useful in the detection of early or low-level responses to pollutants. Although most of the studies on endocrine modulation have been focused on processes involving gonadal steroids, contaminants may target other parts of the endocrine system as well. In this study we examined the adrenocortical stress response and thyroid hormone status in free-living nestling white storks (*Ciconia ciconia*) in relation to heavy metals (zinc, lead, copper, cadmium) and arsenic levels in blood.

**Methods:**

Fieldwork was conducted in an area polluted by the Aznalcóllar mine accident (southwestern Spain) and in a reference site. We used a standardized capture, handling, and restraint protocol to determine both baseline and maximum plasma corticosterone. Circulating levels of thyroxine (T_4_) and triiodothyronine (T_3_) were also measured.

**Results:**

No effects of metals or As were found on baseline corticosterone, but maximum levels of corticosterone were positively related to Pb in both locations. This relationship was stronger in single nestlings than in birds from multiple-chick broods, which suggests a greater impact of Pb on more stressed individuals. Metal pollution did not affect plasma T_4_ or T_3_ levels, although thyroid status differed with location.

**Conclusions:**

Because a compromised hypothalamus–pituitary–adrenal (HPA) function can have far-reaching consequences in terms of altered behavioral and metabolic processes necessary for survival, our results suggest that birds exposed to sublethal Pb levels may be at risk through an altered adrenocortical stress response, and further support the idea that HPA axis-related end points might be useful indicators of metal exposure and potential toxicity in wild animals.

In nature, animals can be used as bioindicators to provide an early warning of potential adverse, contaminant-related effects on organisms or populations themselves, on organisms or populations that prey upon them, and as sentinels for exposure and effects on humans (Fox 2001).

Endocrine assessment is a useful diagnostic tool in the detection of early or low-level responses to pollutants, responses that may precede more significant health problems (Kendall et al. 1998). Although most of the studies on endocrine disruption or modulation have been focused on reproductive problems and behavioral abnormalities related to reproduction, contaminants may target other parts of the endocrine system more commonly than they disrupt processes involving gonadal steroids (Damstra et al. 2002; Hinson and Raven 2006). The hypothalamus–pituitary–adrenal (HPA) axis is an important system that regulates and integrates many physiologic functions in response to environmental stressors (Wingfield and Kitaysky 2002). Activation of the HPA axis during a stress response results in glucocorticoid secretion from the adrenal glands (mainly corticosterone in birds). This, in turn, initiates several important physiologic changes including effects on intermediary metabolism, growth, immune function, and inflammatory responses (reviewed by Kitaysky et al. 2003; Sapolsky et al. 2000). Thyroid hormones— thyroxine (T_4_) and triiodothyronine (T_3_)—also play an important role in metabolism and exert profound effects on avian development (both differentiation and growth) (McNabb 2000).

The impairment of adrenal or thyroid function by contaminant exposure may occur in the absence of gross toxicologic effects and may be critical to developing individuals, with severe consequences such as reduced growth, reduced cognitive capabilities, and impaired immune function (Schantz and Widholm 2001; Smits et al. 2002; Vos et al. 2000). This may result in permanent alterations in the functional capacity of these individuals, with negative long-term consequences to survival and overall fitness (McArthur et al. 1983). Although abnormal glucocorticoid concentrations and impaired functioning of the HPA axis, as well as thyroid modulation, have been linked to exposure to different classes of pollutants (Leatherland 2000; Lorenzen et al. 1999; Mayne et al. 2004, 2005; Smits et al. 2002; Vos et al. 2000; Vyskocil et al. 1990), little is known about the effects of sublethal metal exposure on the adrenal and thyroid systems in free-living birds (Wayland et al. 2002, 2003). Particularly lacking are studies of endocrine modulation in altricial species (Scannes and McNabb 2003).

We conducted a field study to examine the relationships between exposure to heavy metals (zinc, copper, lead, cadmium) and arsenic and the adrenocortical response to stress in wild nestling white storks (*Ciconia ciconia*) hatched in the area affected by the Aznalcóllar mine accident (southwestern Spain) (Grimalt et al. 1999), and in a reference location for comparison. We also evaluated the potential effects of metal pollution on thyroid hormone status in these two groups. Previous studies in the area affected by the mine spill have reported higher genotoxic damage in nestling storks than in controls, with damage having increased 2- to 10-fold between 1999 and 2002 (Pastor et al. 2004). Additionally, nestling storks hatched in the years after the accident had skeletal deformities that were partially related to metal pollution (Smits et al. 2005).

We used a standardized capture, handling, and restraint protocol to assess the adrenocortical response to stress (Wingfield and Kitaysky 2002), and both baseline and maximum plasma corticosterone, as well as plasma T_4_ and T_3_ levels were studied in relation to heavy metals and As concentrations in blood. The effects of other potentially confounding factors affecting endocrine status, such as location, brood size, age, body condition, and sex, were also considered.

## Materials and Methods

### Bird species and study area

The white stork is a large (2,200–4,400 g), long-lived wading bird that breeds from North Africa to northern Europe. At Iberian latitudes, it has a prolonged breeding season (from February to July), with chicks hatching after 33–34 days of incubation and depending on both adults for food and shelter during approximately 75 days before fledging (Cramp and Simmons 1980).

Fieldwork was conducted in two colonies in southwestern and midwestern Spain during spring 2000. The exposed colony was situated in the Dehesa de Abajo (DdA; Puebla del Río, Sevilla, southwestern Spain), in the vicinity of Doñana National Park and only 1 km from the area affected by the Aznalcóllar mine spill in 1998 (Baos et al. 2006). In this colony, white storks breed in open nests at the top of wild olive trees. During the study, about 300 pairs of storks bred at DdA, one of the largest colonies in the western Palearctic (Pastor et al. 2004). The ecologic features of the exposed colony that may influence the results of our study, such as its size (number of breeding pairs), density of breeders, nestlings’ diet [mainly crayfish; see Blas et al. (2005) for details], and its unique location in relation to anthropogenic disturbance (McNabb 2000; Mullner et al. 2004; Silverin 1998), seriously limited the number of colonies we could use as reference. In addition, a reference colony would have to be located far enough away to ensure that there would not be overlap in the use of habitat during the breeding season. Considering all of these requirements, the most suitable colony for a reference was in Cáceres province (CC), about 230 km north of DdA. The reference colony, approximately 50 breeding pairs, was in a scattered oak forest surrounded by crop fields and close to a permanent water source, with crayfish present. Although there were no existing data on metal contamination for this colony, it was located in a natural area far from urban environments and other apparent sources of pollution. Nevertheless, actual metal levels were measured in birds from both colonies for a proper characterization of metal contamination.

### Field procedure and blood sampling

During the first week of June 2000, randomly selected nests of single-chick broods (*n* = 5 in each colony), and two- or three-chick broods (DdA, *n* = 15; CC, *n* = 10) were sampled at both colonies. Blood sampling for hormone and metal residue determinations were performed for either the single nestling or, in the case of two- and three-chick nests, for both the oldest and the youngest birds within a brood (DdA, *n* = 35; CC, *n* = 23 birds). Storks underwent a standardized capture, handling, and restraint protocol known to elicit an increase in circulating corticosterone in birds (Wingfield and Kitaysky 2002). Blood samples (~ 1 mL) were taken from the brachial vein within the first minute after capture, and again at 2, 10, 30, and 45 min [see Blas et al. (2005) for detailed description of the sampling protocol]. A greater amount of blood (2–3 mL) was taken 45 min after capture, transferred to a separate vial (1–2 mL), and frozen immediately at −80°C for determination of heavy metals and As. All birds were individually marked with metal and plastic bands, and their wing-chord length and body mass were measured before they were returned to the nest.

Blood samples for hormone determination were kept on ice until centrifuged (3,000 rpm for 10 min) the same day of capture, and plasma was frozen and stored at −80°C for radioimmunoassay (RIA). Thyroid hormones were quantified in plasma samples taken 2 min postcapture.

The age of nestlings, estimated following the method described by Chozas (1983), ranged between 24 and 59 days. We calculated a body condition index as the individual’s residual value from a reduced major axis regression of log_10_-body mass on log_10_-wing-chord length, following the method of Green (2001). Sex determination was performed through DNA analyses on blood (Ellegren 1996).

This project was approved by the Spanish Animal Care Committee for research involving animals, and the permission to capture nestling white storks at DdA and CC was given by the environmental agencies of the governments of Andalusia and Extremadura, respectively. Birds were treated humanely and with regard for alleviation of suffering during handling.

### Determination of corticosterone and thyroid hormones

We used RIAs to quantify plasma concentrations of corticosterone and thyroid hormones (T_4_, T_3_). In all assays, samples were measured in duplicate, and internal control samples containing known amounts of the hormones were included to ensure that the accuracy of each assay was high and to determine between-assay and within-assay variability. Serial dilutions of samples produced displacement curves that were parallel with each hormone standard curve, indicating that the RIAs were suitable for measuring circulating hormone levels in storks. For corticosterone, samples from the present study were analyzed in the same series of RIAs reported previously (Blas et al. 2005); the assay procedures and parameters were detailed in the same study.

Circulating thyroid hormones are mostly bound to plasma proteins, with only a small amount circulating as free (unbound) hormones. We determined total amounts (i.e., bound plus unbound) of T_4_ and T_3_ in unextracted serum following procedures and reagents described by Smits et al. (2002). These RIAs use 150 μg 8-anilino-1-naphthalene sulfonic acid in each assay tube, and barbital buffer to eliminate interference of serum binding proteins. Samples were diluted 4- to 10-fold prior to use in the assays and measured near the midrange of the standard curves. This was well above the minimum limits of detection (LOD) of 62 pg/mL of plasma for T_3_ and 372 pg/mL of plasma for T_4_. All samples were measured in a single T_3_ or T_4_ RIA; the percent coefficient of variation (%CV) within the T_3_ RIA was 7.4%, whereas the %CV within the T_4_ RIA was 6.3%.

Antiserum and purified hormones used for standards were purchased from Sigma Chemical Company (St. Louis, MO, USA); labeled steroids were purchased from New England Nuclear (Mandel Scientific, Guelph, Ontario, Canada).

### Determination of heavy metals and As

We followed the methodology of Benito et al. (1999) for determination of Pb, Zn, Cu, Cd, and As. Blood samples (1 mL) were prepared using a 30-min ultrasonic extraction with Triton X-100 (Merck Farma y Química, S.A., Valencia, Spain). Zn was analyzed by flow injection-flame-atomic absorption spectrometry using an atomic absorption spectrometer (model 3300) equipped with a flow injection analysis system for atomic spectroscopy (FIAS-400) and an autosampler (AS-90; PerkinElmer Hispania, S.A., Madrid, Spain). Pb, As, Cu, and Cd were measured using a longitudinal AC Zeeman (AAnalyst 600) atomic absorption spectrometer equipped with a transversely heated graphite atomizer and pyrolitic graphite-coated tubes with inserted L’ vov platform (PerkinElmer Hispania, S.A.). Calibration was performed following the method of additions standard curve (Benito et al. 1999). The LODs were 0.23 mg/L for Zn, 2 μg/L for Pb, 24 μg/L for As, 13 μg/L for Cu, and 0.10 μg/L for Cd. Analytical results under the LOD were set to half the respective LOD. The precision as measured by triplicate analysis, expressed as the relative SD, was always < 10%. The range of recovery evaluated by spiking blood samples with each of the elements was 85–115%. We established accuracy for Pb and Cd by analyzing certified reference sample of bovine blood (CRM-195; Institute for Reference Materials and Measurements, Geel, Belgium). For As, and Cu, we used a certified reference sample, TORT-2 (lobster hepatopancreas; National Research Council of Canada, Ottawa, Ontario, Canada). The International Atomic Energy Agency (Vienna, Austria) provided an A-13 reference sample of animal blood for Zn accuracy. The values obtained for all of these elements were consistent with the certified reference values.

### Statistical analyses

To test whether heavy metals and As were related to hormone levels, we analyzed baseline and maximum recorded corticosterone and plasma T_4_ and T_3_ concentrations separately through general linear mixed models (GLMMs) using the MIXED Procedure of SAS (SAS Institute 2001), which simultaneously accounts for random effects, fixed effects, and covariates. When necessary, dependent variables were log_10_-transformed to obtain a normal distribution. Concentrations of heavy metals and As were log_10_-transformed, when necessary, and were included in the models as covariates. We controlled for the potential effects of colony location, brood size, age, sex, and body condition (Blas et al. 2005, 2006) by including them in the models as either fixed factors (colony location, brood size, sex) or covariates (age, body condition). All of the two-way interactions between metals, As, and the rest of the variables were also tested. We considered baseline corticosterone concentration and time (in minutes following capture) to reach the maximum recorded levels as covariates in the analysis performed on the induced response to stress. T_4_ and T_3_ levels were also taken into account as appropriate in the models to test thyroid status. Brood size was categorized as either single- (one-chick nests) or multiple-chick broods (two- or three-chicks nests), because we found no differences in either baseline or stress-induced corticosterone levels between nestlings from two- and three-chick nests (Blas et al. 2005). To avoid pseudoreplication derived from the lack of independence in values from nestlings within the same brood and sharing the same colony, we included the nest as a random variable (nested into colony; Littell et al. 1996).

The models were constructed by a stepwise forward procedure, testing the significance of each variable and adding only the variable that resulted in a better fit to the model (Baos et al. 2006). Final (i.e., minimum adequate) models retained only those terms showing a significant effect on hormone concentrations at a > 5% rejection probability. All tests were two-tailed.

## Results

### Heavy metals and As levels

Concentrations of Cu, Zn, and As did not differ between locations (Table 1). Pb levels were significantly higher in nestling storks from the reference colony (Table 1), although only one individual from CC (4.3 %; *n* = 23) and none from DdA showed Pb concentrations > 200 μg/L, the value reported to cause sublethal effects in birds (Franson et al. 1996). All blood Cd concentrations were < LOD (0.10 μg/L) in both locations; therefore, Cd was not considered in subsequent analyses.

### Relationships between hormone concentrations, heavy metals. and As levels

#### Baseline corticosterone

We found no significant relationships between heavy metals or As and baseline corticosterone levels, either tested alone (*F* < 2.78; *p* > 0.110) or through interactions with colony location, brood size, age, sex, and body condition (*F* < 1.60; *p* > 0.222). Only colony location (*F*_1,32_ = 35.91; *p* < 0.0001) and brood size (*F*_1,23_ = 6.03; *p* = 0.022) were related to baseline levels of corticosterone, with birds from CC showing higher hormone concentrations (mean ± SD, 30.6 ± 11.6 ng/mL; *n* = 23) than those from DdA (14.1 ± 7.3 ng/mL; *n* = 35), and single nestlings showing higher levels (32.3 ± 17.3 ng/mL; *n* = 10) than birds from multiple-chick broods (18.2 ± 9.4 ng/mL; *n* = 48).

#### Stress-induced corticosterone

**Figure 1:**
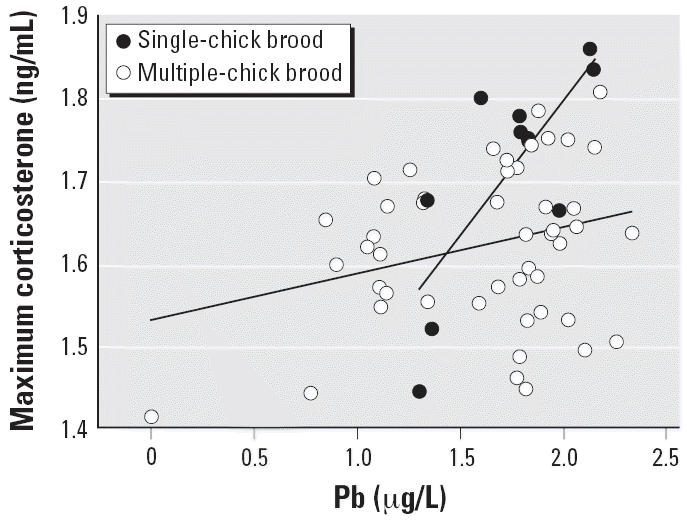
Relationships between Pb levels (log-μg/L) in blood and the stress-induced response (i.e., maximum corticosterone concentration, log-ng/mL) in nestling white storks from single chick-broods (*n* = 10) and multiple-chick broods (*n* = 48) after 45 min of handling and restraint (from both colonies).

Maximum corticosterone concentration was positively related to blood Pb levels (*F*_1,20_ = 10.93; *p* = 0.004; Table 2) in both colony locations (interaction term, Pb ×colony, *F*_1,19_ = 0.58; *p* = 0.456), and singletons had higher levels of corticosterone (53.3 ± 14.6 ng/mL; *n* = 10) than nestlings from multiple-chick broods (42.9 ± 9.3 ng/mL; *n* = 48; *F*_1,20_ = 5.36; *p* = 0.031; Table 2). The interaction term Pb ×brood size was also significant (*F*_1,20_ = 7.30; *p* = 0.014), suggesting that Pb had a greater effect on the stress-induced corticosterone of single nestlings than on those of multiple-chick broods (Table 2, Figure 1). Neither Cu, Zn, nor As levels alone or as part of interaction terms showed any significant relationship with maximum corticosterone values (*F* < 2.80; *p* > 0.113). Age was positively related to the stress-induced corticosterone secreted after applying our handling and restraint protocol (*F*_1,20_ = 6.74; *p* = 0.017; Table 2). The final model also included a positive association between baseline and maximum corticosterone concentrations (*F*_1,20_ = 4.69; *p* = 0.043; Table 2), but neither sex, body condition, nor time elapsed to reach the maximum hormone concentration showed a significant effect.

#### Thyroid hormones

The analysis of plasma T_3_ levels revealed that neither heavy metals, As concentrations, brood size, sex, age, body condition, nor any of the interaction terms accounted for differences in hormone levels (*F* < 3.34; *p* > 0.085). Plasma T_4_ concentrations were positively related to T_3_ (*F*_1,21_ = 13.20; p = 0.002) as expected, given that T_4_ is the precursor of active T_3_. Although absolute T_3_ levels were similar between colonies (CC, 1.05 ± 0.3 ng/mL, *n* = 23; DdA, 1.09 ± 0.4 ng/mL, n = 35), when controlling for T_4_ the model showed a significant effect of location (*F*_1,31_ = 4.67; *p* = 0.039).

Regarding the variables affecting T_4_ concentrations, only colony location had a significant effect, being higher in DdA (9.5 ± 3.2 ng/mL; *n* = 33) than in CC nestlings (5.3 ± 1.5 ng/mL; *n* = 22; *F*_1,31_ = 40.41; *p* < 0.0001). No remaining variables or interactions were significant in the final model accounting for variation in plasma T_4_ (*F* < 3.19; *p* > 0.089).

**Figure 2:**
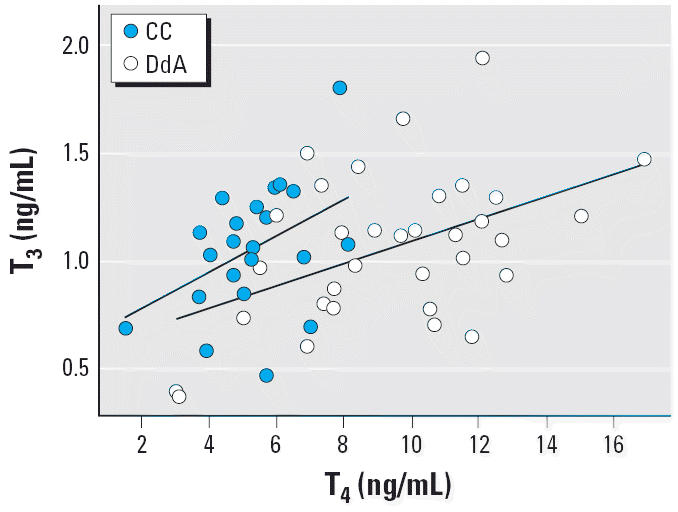
Relationship between plasma T_4_ and T_3_ concentrations in nestling white storks from CC (reference) and DdA (exposed) colonies.

Both models suggest that for a given level of T_3_ (which was similar between colonies), birds from DdA had higher levels of the precursor T_4_ (Figure 2).

## Discussion

### Heavy metals and As levels

Blood levels of essential metals Cu and Zn were similar between colony locations, and generally lower than those reported in bird populations from other metal-polluted areas (Beyer et al. 2004; Van Eeden and Schoonbee 1996). Contrary to our expectations, nestlings from the reference colony (CC) had higher blood Pb levels than those sampled in DdA. Nevertheless, Pb levels > 200 μg/L, the value reported to cause sub-lethal effects in birds (Franson 1996), were found in only one nestling from CC, with the remaining individuals from both CC and DdA having “background” concentrations. When working with wildlife, one major difficulty rests with the establishment of an absolute control condition, when in fact the best option may be a reference population for comparison. Although no previous studies on any pollutant had been conducted in the reference location, the stork colony was in a natural area far from apparent sources of contamination. This result supports the argument of Norris (2000) that the widespread distribution of many potential endocrine-modulating substances makes it difficult, if not impossible, to find a true control population. Blood levels of As were found at concentrations below those reported in birds from contaminated areas (Burger and Gochfeld 1997) and were similar between the two sampled locations.

### Relationships between hormone concentrations, heavy metals, and As levels

Our results showed a positive association between Pb and the stress-induced response elicited after 45 min of handling and restraint regardless of colony location. Of particular importance is that this relationship occurred at Pb levels below those normally associated with detrimental health effects in birds (Franson 1996). Recent experimental and field studies on passerines exposed to Pb have reported no effect on the stress response (Eeva et al. 2003, 2005; Snoeijs et al. 2005); however, these studies measured corticosterone levels just after capture (i.e., baseline concentrations) for which we also did not find a significant relationship.

Lead ranks second on the list of top 20 hazardous substances from the 2005 CERCLA (Comprehensive Environmental Response, Compensation, and Liability Act) Priority List [Agency for Toxic Substances and Disease Registry (ATSDR) 2005]. The widespread use of lead arsenate as a pesticide in the early 20th century left a broad legacy of soil and groundwater contamination. Recent or past lead-shot hunting, mining, and smelting activities and industrial effluents are other sources of Pb that have made this element one of the most common metals in contaminated ecosystems (ATSDR 1999). Exposure to Pb is associated with neurobehavioral, hematologic, nephrotoxic, and reproductive effects in birds and other animals (Scheuhammer 1987). Experimental studies in mammals have shown that both adult and developmental Pb exposure elevate baseline corticosterone levels (Cory-Slechta et al. 2004; Der et al. 1977; Vyskocil et al. 1990); however, similar effects have not been reported on stress-induced response in either mammals or avian species. Therefore, we cannot discount other factors potentially correlated with Pb (e.g., pesticide exposure) that may contribute to some degree to the positive relationship we found in storks between Pb and the stress response. Experimental studies are needed to establish a cause–effect relationship.

In the present study we also found that the association between Pb and the stress-induced response was greater for birds from single-chick broods than for those from multiple-chick broods. In a previous article (Blas et al. 2005), we reported that single nestlings were reared in nests that experienced brood reduction, which suggested lower parental quality. Reduced attendance by young or inexperienced parents may lead singletons to suffer from environmental stressors other than Pb (e.g., a greater exposure to harsh weather conditions). This in turn may explain both their higher levels of maximum corticosterone (Blas et al. 2005) and the stronger relationship between their induced response to stress and Pb. In a recent experimental study on rats, Cory-Slechta et al. (2004) provide evidence that Pb exposure and stress interact. Furthermore, the fact that both stress and Pb target brain mesocorticolimbic dopaminergic systems (e.g., Barrot et al. 2000; Cory-Slechta et al. 1997) provides a biological basis for such an interaction. Di Giulio and Scanlon (1985) reported that the highest plasma and adrenal concentrations of corticosterone were observed in ducks receiving the highest level of dietary cadmium when they were food-restricted. Similarly, Wayland et al. (2002) found significant correlations between renal Cd concentration and the stress response of female common eiders (*Somateria mollissima borealis*) during the incubating period, when they are reported to fast, whereas no effects were detected in prenesting females. In this sense, although it has to be interpreted with caution because of the small sample size, our result would support the argument that contaminants acting in concert with other stressors may have a greater impact on individuals or populations than would be elicited by either the contaminants or other stressors acting alone.

Although temporary increases in corticosterone are believed to be beneficial, serving to redirect individuals’ behavior and physiology to foraging or self-maintenance and to enhance assimilation of energy during the stressful period, chronic or frequent activation of the stress response can be deleterious, resulting in growth suppression, reproductive dysfunction, and immunosuppression (Wingfield and Kitaysky 2002). The brain is also a target of the adverse effects of prolonged elevation of glucocorticosteroids (Kerr et al. 1991), with deleterious effects that may include impairment of cognitive function (Kitaysky et al. 2003). Thus, a compromised HPA function by Pb, alone or in conjunction with other environmental stressors, could have far-reaching consequences in terms of altered behavioral and metabolic processes necessary for survival.

Few studies have reported the effects of metal pollution on the homeostasis of thyroid hormones in birds, especially in natural settings (Leatherland 2000). In the present study, neither heavy metals nor As were related to circulating levels of thyroid hormones. However, lower levels of T_4_ were found in the location with higher concentrations of Pb (CC, the reference site). Although this result may suggest that circulating levels of T_4_ are susceptible to Pb exposure in nestling storks, colony location and not Pb levels accounted for differences in plasma T_4_. The studies reporting Pb effects on circulating thyroid hormones have focused on heavily exposed subjects, often with other manifestations of lead poisoning (Robins et al. 1983; but see also Refowitz 1984). Thus, a possible explanation for the lack of significant relationships between Pb and plasma thyroid hormones is that metal levels measured in nestling storks were too low to exhibit a significant correlation. Alternatively, location-dependent intrinsic (e.g., genetic) or extrinsic (e.g., environmental) factors other than variation in Pb, including pollutants other than those analyzed (e.g., polychlorinated biphenyls, dioxins, pesticides) could potentially account for our results.

## Conclusions

The present study provides, to our knowledge, the first evidence that Pb may adversely affect the HPA axis function of wild birds. The association we found occurs at Pb levels below those reported to cause sublethal effects in avian species, and further supports the idea that HPA axis–related end points may be useful indicators of metal exposure and potential toxicity in wild animals. Experimental studies in this regard are strongly encouraged.

Our results indicate that a simple handling and restraint protocol may be a better approach to assess the HPA axis function than a single measurement taken after capture. Plasma corticosterone is a static measure by itself and does not provide information concerning the dynamic nature of the system (Hinson and Raven 2006; Norris 2000). This work also supports the idea that greater attention to the problems arising from interactions between different types of stressors must certainly be considered, especially when working in the wild, given that such a scenario (and not the exposure to isolated sources of stress) constitutes the environmental reality.

The persistence of Pb, combined with the sensitivity of the HPA axis to contaminant insult, suggests that exposure to this metal even at sublethal levels may cause delayed functional deficits later in development. Endangered species and/or populations exposed to additional stress factors (e.g., nutrition deficiency, parasitism) would be of special concern. Further field studies involving birds from these and other metal-polluted sites are required to determine the extent to which the corticosterone response systems of wild birds are affected by exposure to Pb as well as the long-term effects on survival and overall fitness.

Thyroid status was not related to metal pollution, although we found differences between colony locations that deserve further investigation.

## Figures and Tables

**Table 1:** Mean ± SD (range) of heavy metals and arsenic (μg/L or mg/L wet weight) in blood of nestling white storks according to colony location.

Colony location^a^	Cu (μg/L)	Pb (μg/L)	Zn (mg/L)	As (μg/L)
CC (reference)	359.5 ± 55.5 (233–451) *n* = 23	90.7 ± 51.0 (6–214) *n* = 23	2.8 ± 0.5 (1.8–3.8) *n* = 22	48.6 ± 46.8 (ND–205) *n* = 20
DdA (exposed)	357.2 ± 41.1 (247–426) *n* = 34	44.4 ± 33.6 (ND–126) *n* = 35	2.9 ± 0.4 (2.1–3.7) *n* = 35	34.5 ± 51.4 (ND–248) *n* = 32

ND, below the LOD (see “Materials and Methods” for details).

a Results of GLMMs testing for differences between colony locations using normal error and identity link function (nest = random factor): Cu, *F*_1,33_ = 0.05, *p* = 0.829; Pb, *F*_1,33_ = 7.59, *p* = 0.010; Zn, *F*_1,33_ = 0.31, *p* = 0.579; As, *F*_1,31_ = 3.11, *p* = 0.088.

**Table 2:** Summary of results from the GLMM explaining stress-induced response (i.e., maximum cortico-sterone concentration after 45 min of handling and restraint) in nestling white storks (*n* = 58 birds).

Effect	Estimate	SE	*F-*Value (df)	*p*-Value
Intercept	16.660	7.666	2.17^a^ (32)	0.037
Baseline corticosterone	0.246	0.113	4.69 (1,20)	0.043
Age	0.349	0.134	6.74 (1,20)	0.017
Brood size	−38.160^b^	16.484	5.36 (1,20)	0.031
Pb (log)	3.596	2.892	10.93 (1,20)	0.004
Pb (log) × brood size	25.941^b^	9.601	7.30 (1,20)	0.014
All other effects			< 2.80	> 0.113

df, degrees of freedom. The estimated effect, SE, *F*-values, and associated probabilities are shown for those variables that significantly improved the fit of the model.

a *t*-Test.

b Estimate corresponds to brood size = 1.
